# Traffic-Related Air Pollution and Childhood Asthma

**DOI:** 10.1289/ehp.1002224

**Published:** 2010-07

**Authors:** Francesco Cetta, Marco Sala, Marina Camatini, Ezio Bolzacchini

**Affiliations:** PAT Geriatric Institute, Milan, Italy, E-mail: cetta@unisi.it; Department of Pediatrics, University of Milan, S.Paolo Hospital, Milan, Italy; Department of Environmental Science University of Milano Bicocca, Milan, Italy

We congratulate Clark et al. (2010) for their interesting article concerning traffic-related air pollution and asthma in children. They examined early-life (*in utero* and during the first year of life) exposure to traffic-related air pollution in a large population-based study (a nested case–control study including nearly 3,500 children). The authors found an association between elevated early-life exposure to traffic-related air pollution and a higher risk of asthma in preschool-aged children. However, Clark et al. (2010) address aspects, raise questions, and give results that deserve further comment—both concerning specific items and general “structural” criteria used in epidemiological studies on adverse health effects from air pollution.

Recent reports have suggested that individual susceptibility could play a previously unsuspected role in the occurrence of diseases (Cetta et al. 2009a), perhaps a role greater than that of the intrinsic toxicity of pollutants (Cetta et al. 2009b). This could explain, at least in part, why it is so difficult to determine a precise threshold concentration that is harmful or safe for each individual (Cetta et al. 2007). But this is just one side of the question.

The main question is that, in the absence of adequate and specific markers of exposure, effect and susceptibility, the linear dose and effect model, and the concomitant pollutant concentration and disease occurrence relationship cannot explain the complexity of the phenomenon of host–particle interactions. In particular, initial cell alterations (e.g., oxidative stress, DNA adduct formation) rarely turn into permanent tissue damage and evident disease because of host repair and defence mechanisms.

In their article Clark et al. (2010) noted another important aspect that should be considered when comparing pollutant concentrations with the burden of deleterious effects, both at the individual and population levels: acute effects of peak concentrations of pollutants that lead to acute admission to hospital and the chronic damage that causes long-term effects. In fact, we should consider both of these as separate entities. However, we also should consider the effect of air pollution on newborns, which greatly depends on individual susceptibility—either congenital or acquired. This could play a major role in future outcomes and shed new light on the peculiar pathophysiological mechanisms of most pollution-related diseases. Clark et al. (2010) correctly outlined the asynchronism and the delay (0–4 years) between the initial pathogenetic exposure to pollutants (*in utero* or during the first year of life) and the occurrence and detectability of clinically relevant asthma. This further adds to the complexity of host-particle interactions.

There are three issues that should be taken into account in developmental epidemiology studies such as that by Clark et al. (2010). First, epidemiological studies that concomitantly evaluate pollutant concentration and detectable diseases or hospital admissions usually neglect the perinatal damage in fetuses and newborns, which is not immediately detectable but is a delayed manifestation.

Second, perinatal damage from air pollution deserves further attention and detailed analysis because it includes fetal malformations, birth defects, and developmental alterations of newborns. Injury from perinatal air pollution exposure could also be responsible for the increased proportion of unsusceptible individuals who, because of their exposure to pollutants during the susceptibility window and because of epigenetic alterations due to environmental factors, will become susceptible. Epigenetic alterations could also transfer this susceptibility to future generations, leading to individuals developing not only asthma at 4 years of age but also respiratory, cardiovascular or systemic diseases ≥ 20 years later.

Third, clinical and pathophysiological details are not “details” but basic issues and questions—still unsolved— that should be primary goals for future research. They are critical to improving design of epidemiologic studies and to selecting appropriate models, which should also include biological and pathophysiological parameters and variables because they significantly affect clinical outcomes.
