# Errata

**Published:** 2010-09

In the letter “Traffic-Related Air Pollution and Childhood Asthma,” published in the July issue of *EHP* ( Cetta et al. 2010. Environ Health Perspect 118:A283–A284), Marina Camatini and Ezio Bolzacchini (Polaris Research Center, Department of Environmental Science, University of Milano Bicocca, Milan, Italy) were listed as authors. However, they have notified *EHP* that they were not informed about the letter and that their names were included without their permission. Both Bolzacchini and Camatini have requested to have their names removed; therefore, *EHP* will honor their request and remove their names from the online versions of the letter.

